# Novel Bunyavirus in Domestic and Captive Farmed Animals, Minnesota, USA

**DOI:** 10.3201/eid2002.131790

**Published:** 2014-02

**Authors:** Zheng Xing, Michael Murtaugh

**Affiliations:** College of Veterinary Medicine, University of Minnesota at Twin Cities, St. Paul, Minnesota, USA (Z. Xing, M. Murtaugh);; Medical School, Nanjing University, Nanjing, China (Z. Xing)

**Keywords:** bunyavirus, phlebovirus, Heartland virus, severe fever with thrombocytopenia syndrome virus, sftsv, animals, infection, viruses, Minnesota, USA, large mammals, novel bunyavirus

**In Response**: We welcome the critiques of Nasci, et al. (*1*), who may have misinterpreted the point of our Dispatch. Regarding identification of Heartland virus (HLV) in farm animals, in our article (*2*), we stated that “the viruses detected in this region are most likely HLV or close relatives of HLV,” which indicates that the exact identification of the viruses in the animals in Minnesota will not be confirmed until the viruses are isolated and/or the genomic sequence data are available. 

The underlying data were obtained with an ELISA specific to severe fever with thrombocytopenia syndrome virus (SFTSV). The conclusion was based on our knowledge, at the time our manuscript was submitted, that in North America no other known phleboviruses of this expanded Uukuniemi group that contains SFTSV and HLV were reported to be cross-reactive with SFTSV. When tested by using our reagent, SFTSV was not cross-reactive with Rift Valley fever virus. Related phleboviruses of this group (e.g., Bhanja, Palma, Forecariah, and Kismayo viruses) have not been reported in North America (*3*). Phleboviruses of this group, such as Murre virus and RML-105355 virus, and Sunday Canyon virus, were isolated in Alaska and Texas, respectively, but are not cross-reactive with SFTSV (*4*). 

Other bunyaviruses in North America (e.g., Cache Valley virus and California serogroup viruses) are distantly related and have ≈11% amino acid sequence homology to SFTSV. The recently characterized Lone Star virus appears to be the closest relative to SFTSV and HLV and may cross-react with SFTSV and HLV, as also suggested by Nasci et al., but this virus is apparently known only from 1 isolate obtained in 1967 (*5*). These data suggest that SFTSV is not serologically cross-reactive with the known Unkuniemi group viruses that are currently being transmitted in North America. Our report shows that tickborne phleboviruses, which are closely related to SFTSV and HLV, may be more generally distributed in the midwestern United States and emphasizes the need to substantiate our serologic evidence with virus isolation and genomic characterization, which are underway.
